# Greek students’ attitudes, perception and knowledge regarding generic medicines in times of economic crisis: a cross-sectional study

**DOI:** 10.1186/s12909-018-1379-8

**Published:** 2018-11-15

**Authors:** Philippe J. Domeyer, Vasiliki Katsari, Pavlos Sarafis, Vassilis Aletras, Dimitris Niakas

**Affiliations:** 1 School of Social Sciences, Hellenic Open University, Patras, Greece; 2 Nursing Department, Cyprus University of Technology, Limassol, Cyprus; 3 Department of Business Administration, University of Macedonia, Thessaloniki, Greece; 4 Μedical School, National and Kapodistrian University of Athens, Athens, Greece

**Keywords:** Generics, Attitude, Knowledge, Students, Greece, Drug substitution, Trust in generics, Fiscal impact, State audit, Quality of generics

## Abstract

**Background:**

The penetration of generic medicines in the pharmaceutical market is influenced, among others, by the consumer’s attitude upon them. The attitude of students in health management and recent alumni is particularly important, as they constitute tomorrow’s policymakers. The aim of our study was to assess their attitude, perception and knowledge towards generic medicines.

**Methods:**

A cross-sectional study was undertaken, involving students in Health Management and recent alumni. The ATtitude TOwards GENerics (ATTOGEN) validated questionnaire was used, which consists of 18 items, yielding 6 scales (trust, state audit, knowledge, drug quality, drug substitution and fiscal impact), with all item responses expressed on a 5-point Likert scale and higher scores denoting greater disagreement. Correlation coefficients were computed and independent sample tests were performed using non-parametrical statistical methods.

**Results:**

A total of 1402 students were interviewed, with a female predominance (62.88%). The mean (SD) scores for the six scales of the ATTOGEN questionnaire were: Trust: 2.877 (0.940), State audit: 3.251 (0.967), Knowledge: 1.537 (0.688), Drug quality: 2.708 (0.971), Drug substitution: 3.828 (1.127) and Fiscal impact: 2.299 (0.860). Trust over generics was statistically significantly associated with all ATTOGEN scales (all *p* < 0.001). In addition, the increased level of knowledge about generics was associated with recognition of the generic medicines’ quality equivalence (*p* < 0.001) and positive fiscal impact (*p* = 0.018). Pharmacists declared having a superior knowledge of generic medicines, being more satisfied with the information they receive about them and strongly believing in drug substitution (*p* < 0.001). Comparatively to other professionals, pharmacists also indicated substantial differences between branded and generic medicines more often (*p* < 0.001). They also argued to a greater extent that generic medicines were invented and promoted to resolve the financial crisis of social security institutions at the expense of citizens (*p* < 0.001).

**Conclusions:**

This study demonstrated a mixed attitude of students regarding generic medicines. Trust and knowledge emerged as key factors shaping the students’ attitude towards generics. Among students, pharmacists exhibited a distinct response pattern. This study underlines the importance of addressing and correcting health management students’ misbeliefs about generics’ quality and utility.

**Electronic supplementary material:**

The online version of this article (10.1186/s12909-018-1379-8) contains supplementary material, which is available to authorized users.

## Background

Generic medicines are manufactured by companies other than the manufacturer of the originator medicine, after various kinds of commercial protections granted to the latter have expired. According to the World Health Organization, generic medicines hold the following three characteristics: i) they are intended to be interchangeable with an innovator product, ii) they are manufactured without license from the innovator company and iii) they are marketed after the expiration date of the patent or other exclusive rights [1]. Generic medicines differ from biosimilars in terms of size, complexity of the active substance and the manufacturing process [2]. In order to penetrate the pharmaceutical market, they are competitively priced against their brand name counterparts, while ensuring bioequivalence. Generic medicines thus seem to be a cost-effective solution for healthcare systems, especially in light of the severe economic crisis, as their reduced cost can decrease private and public drug expenditure and can contribute to reallocation of funding towards other needs [3].

Despite the obvious cost-effectiveness of generic medicines, their penetration in the pharmaceutical market is often limited. Greece, albeit a country under severe economic surveillance, performs poorly in terms of generic medicines penetration. Strikingly, the rate of penetration 12 months after the loss of exclusivity in Greece is the lowest among the former 12-European Union member states (33%). Even worse, the degree of generic penetration measured by the share of generic volume in total volume at 24 months after the loss of exclusivity is again the lowest in Greece (9.1%), suggesting an important time delay to generic entry [4]. In addition, in 2015 the volume share of generics in the total Greek pharmaceutical market reached less than half the OECD27 average value (24% vs 52%, respectively) [5].

This apparently limited penetration of generic medicines may be highly influenced by a number of factors, mainly institutional ones. In particular, the effectiveness of policies implementing generic use may depend on whether physicians or patients may overrule generic substitution, the motivation or obstacles for pharmacists to dispense generic over branded products and the price difference between brand name and generic products [4]. Another recognized influential factor is the consumers’ attitude, perception and knowledge towards generic medicines, which may in turn be influenced, among others, by advertising campaigns and information dissipated from the mass media [6] as well as consumers’ inherent beliefs. Indeed, both healthcare providers and patients face generic medicines with skepticism [7]. Generics are thought to be inferior medicines [8], although no such tangible proof exists [9], perhaps because people often perceive “generic” to be identical to “cheap”, thus indicating inferior quality [8], although this is not always true [10]. In fact, in a study involving students, it was shown that patients complain about reduced effectiveness and increased medication-related side-effects when drug substitution from branded to generics occurs, which may partially be due to a loss of associated placebo effects and enhanced nocebo effects [11]. Nevertheless, patients with higher confidence in the healthcare system and higher educational and socioeconomic background seem to show less concern about generic substitution [12, 13].

In order to boost the use of generic medicines, healthcare policy makers should update their strategy. As a result, educational activities regarding the benefits of generics use should be targeted towards students of M.Sc. programs in Health Management, who are the future policymakers. Although a considerable number of studies have been published regarding healthcare students’ knowledge, attitudes and perceptions concerning generic medicines [14–24], none of them used a validated tool and none has emphasized on students and alumni of M.Sc. programs of Healthcare Management. The present study attempts to fill this important gap in the literature.

## Methods

### Study design, population and sampling

The study sample of this cross-sectional study consisted of Greek students and recent (up to one year from graduation) alumni of the M.Sc. program in Health Management of the Hellenic Open University (H.O.U.). This program requires students to successfully complete 4 modules plus a thesis project. The choice of this particular M.Sc. program was dictated by the following facts:

1. The H.O.U. ranks first in the number of students in Greece. Therefore, the large pool of students and alumni would ensure increased statistical power.
2. Distant learning and e-learning enables the recruitment of students from a broad range of the Greek population, encompassing different age, economic and sociodemographic groups, therefore increasing the generalizability of the study’s results.
3. The familiarity of all participants with electronic media enabled the use of an electronic platform for fast and flawless completion of the questionnaire.

### Study tool

For the purposes of our study the ATtitude TOwards GENerics (ATTOGEN) questionnaire, an already validated instrument published elsewhere [23] was used, exploring respondents’ attitudes towards generic medicines. This instrument consists of 18 items, with all item responses expressed on a 5-point Likert scale (1 = totally agree, 2 = partially agree, 3 = neither agree nor disagree, 4 = partially disagree, 5 = totally disagree). During the validation procedure 6 scales were created, accounting for 96.1% of the total variance: Scale 1 (“Trust”, 4 items), with lower values denoting increased mistrust to generic medicines; Scale 2 (“Drug quality”, 3 items), with lower values supporting same drug quality of generic and brand name medicines; Scale 3 (“State audit”, 3 items), with lower values indicating increased confidence that Greek and European authorities may detect irregularities in generic medicines production; Scale 4 (“Fiscal impact”, 3 items), with lower values representing a stronger belief in generic medicines’ positive economic impact; Scale 5 (“Knowledge”, 3 items), with lower values reflecting increased knowledge regarding generic medicines; Scale 6 (“Drug substitution”, 2 items), with lower values designating a more positive view towards allowing not only doctors but also pharmacists to perform substitution of brand-name with generic medicines. The instrument’s internal consistency has been shown to be high (0.871); the same applies for most of its subscales [23]. The instrument’s confirmatory factor analysis has also shown that the proposed model provided a good fit (RMSEA = 0.050, RMSR = 0.048, TLI = 0.948, CFI = 0.960) [23]. A separate analysis was performed on questions regarding the use of generic medicines that were included in the original but not in the final version of the instrument [23].

### Data collection method

Details regarding the instrument administration and ethics have already been described [24]. In brief, the instrument was uploaded to Google Drive (Google Inc., California, USA) and a web link was sent to all participants. To submit the questionnaire, they had to complete all fields. Monthly reminders were sent to maximize the response rate. Anonymity was ensured and ethics approval was obtained by the Institutional Review Board.

### Data analysis method

Normality of data was tested using Kolmogorov-Smirnov test. To analyze group differences, non-parametric tests were used, including the chi-square test, when associations between two categorical values were tested, as well as the Wilcoxon rank-sum test and the Kruskal-Wallis rank test, when two or more than two related samples where compared, respectively. Spearman’s correlation coefficient was used to evaluate the monotonic relationship between continuous or ordinal variables. Two-tailed *p* values < 0.05 were considered statistically significant. When multiple (k) comparisons were performed, the Bonferroni correction was adopted, thereby lowering the threshold for statistical significance to 0.05/k. Statistical analysis was performed using Stata version 13.1 (Stata Corp., Texas, USA).

## Results

Among 1402 students that were contacted, 986 completed the online survey (response rate = 70.32%). The mean age was 39.8 (7.11 years, with a female predominance (62.88%). The majority of the students were married (63.69%). Most of them were health professionals (80.93%), among which 33.83% where doctors, 24.31% were nurses, 4.51% were pharmacists, 4.01% were dentists and 33.34% were other health professionals. Finally, only 4.87% were unemployed. The mean (SD) scores for the six scales of the ATTOGEN questionnaire were: scale 1 (Trust): 2.877 (0.940), scale 2 (State audit): 3.251 (0.967), scale 3 (Knowledge): 1.537 (0.688), scale 4 (Drug quality): 2.708 (0.971), scale 5 (Drug substitution): 3.828 (1.127) and scale 6 (Fiscal impact): 2.299 (0.860). Table 1 presents the descriptive statistics of items that were used in the original but not in the final version of the ATTOGEN questionnaire and where consequently not included in any of the above scales.

**Table 1:** Descriptive statistics of items included in the original version of the ATTOGEN questionnaire only

Item	Item description (ordinal variables)	Mean (SD) score	Median score (IQR)^a^
I	“I am satisfied with the information I have regarding generic medicines”	2.781 (1.369)	3 (2)
II	“I have noted substantial differences between brand-name and generic medicines”	3.164 (1.107)	3 (1)
III	“I am skeptical about generic medicines because of their lower price”	3.647 (1.074)	4 (1)
IV	“I believe that generics were invented and promoted in order to resolve the financial crisis of social security institutions at the expense of citizens”	3.267 (1.251)	3 (2)
V	“Among two generic medicines, I would trust more the one that is manufactured in Greece”	2.216 (0.967)	2 (2)
	Item description (categorical variables)	N (%)	
VI	“I know if my current medications include generic medicines”		
	*Yes*	375 (38.03%)	
	*No*	24 (2.43%)	
	*I am not taking medications at the moment*	587 (59.53%)	

^a^*IQR* Interquartile range

Inter-scale correlations of the ATTOGEN questionnaire are shown in Table 2.

**Table 2:** Inter-scale correlations^a^

Scale	1	2	3	4	5	6
1 (Trust)	–					
2 (State audit)	−0.276^§^	–				
3 (Knowledge)	−0.262^§^	0.032	–			
4 (D quality)	−0.620^§^	0.361^§^	0.352^§^	–		
5 (Drug substitution)	−0.232^§^	0.034^§^	0.172	0.208^§^	–	
6 (Fiscal impact)	−0.376^§^	0.075^§^	0.371^¶^	0.433^§^	0.235^§^	–

^a^Calculated with Spearman’s correlation coefficient

§ *p* < 0.001, ¶ *p* = 0.018

The “Trust” scale was statistically significantly associated with all other ATTOGEN scales, indicating that a higher trust score (i.e. increased trust over the generics) was related to increased belief in the state’s audits abilities to control generics production, in generics drug quality and positive fiscal impact, in drug substitution and an increased knowledge regarding generics (all *p* < 0.001). In addition, students more knowledgeable about generics seemed to recognize the generic medicines’ quality equivalence (rho = 0.361, *p* < 0.001) and positive fiscal impact (rho = 0.075, *p* = 0.018). The perception of the fiscal impact of generic medicines was statistically significantly associated with the rest of the ATTOGEN questionnaire scales (all *p* < 0.001).

Inter-item associations for items I-V included only in the original version of the ATTOGEN questionnaire were strongly statistically significant in most cases (Table 3).

**Table 3:** Inter-item correlations for items I-V*

Items	I	II	III	IV	V
I	–				
II	0.059	–			
III	−0.227^§^	0.149^§^	–		
IV	−0.265^§^	0.202^§^	0.474^§^	–	
V	0.186^§^	0.016	−0.056	−0.029	–

* Calculated with Spearman’s correlation coefficient, § *p* < 0.001

In particular, satisfaction with generic medicines seemed to be strongly correlated with decreased skepticism about them, increased trust on those manufactured in Greece and a weaker belief that they were invented and promoted to resolve the financial crisis of social security institutions at the expense of citizens (all *p* < 0.001). In addition, this latter belief was associated with skepticism about generic medicines because of their lower price and with having noted substantial differences between brand-name and generic medicines (all *p* < 0.001). Furthermore, students who declared knowing if their medications involved generic medicines (item VI) were also more likely to report: i) higher satisfaction with the information they have regarding generic medicines (item I, *p* < 0.001), ii) substantial differences between brand-name and generic medicines (item II, *p* < 0.001), iii) more skepticism about generic medicines because of their lower price (item III, *p* = 0.001) and iv) a better knowledge about generic medicines (scale 3, *p* < 0.001).

Most item-scale associations between items I-IV and scales 1 to 6 were also strongly statistically significant (Table 4), indicating that the satisfaction regarding generics, the notice of differences between brand-name and generics, the skepticism about generics because of their lower price and the belief that generics were invented and promoted in order to resolve the financial crisis of social security institutions are key elements related to the ATTOGEN scales.

**Table 4:** Item-scale correlations for items I-V and scales 1–6*

Items/scales	1	2	3	4	5	6
I	−0.193^§^	0.132^§^	0.441^§^	0.317^§^	0.028	0.084^¶^
II	0.434^§^	−0.110§	0.040	−0.310^§^	− 0.148^§^	−0.241^§^
III	0.512^§^	−0.085^¶^	−0.334^§^	− 0.323^§^	−0.102^¶^	− 0.115§
IV	0.525^§^	−0.093	−0.320^§^	− 0.367^§^	−0.113§	− 0.121§
V	−0.011	0.041	0.198^§^	0.017	−0.060	−0.035

* Calculated with Spearman’s correlation coefficient, § *p* < 0.001, ¶ *p* < 0.01

Table 5 presents the statistically significant associations between sociodemographic factors and the questionnaire’s scales. [Table 5] In addition, older students, men and pharmacists expressed a stronger preference to generic medicines manufactured in Greece (rho = − 0.144, all *p* < 0.001) and seemed more satisfied with the information they have regarding generic medicines (rho = − 0.145, all *p* < 0.001). Furthermore, compared to other professionals, pharmacists noticed substantial differences between brand name and generic medicines and believed that generics were invented and promoted to resolve the financial crisis of social security institutions at the expense of citizens, despite their better score on the “knowledge” scale; differences with other professionals were all statistically significant (all *p* < 0.001). This last opinion was also expressed more favorably by women (*p* < 0.001), whose negative opinion regarding generic medicines, compared to men, was further intensified by the fact that they appeared more skeptical about generic medicines because of their lower price (*p* < 0.001). Finally, among students on medications, older students and health scientists seemed to have a better knowledge if their current medications involved generic medicines (*p* = 0.014 and < 0.001, respectively).

**Table 5:** Statistically significant associations between sociodemographic factors and the questionnaire’s scales

Scales	Associated factors	Mean (SD)*	Median (IQR) **	Spearman’s rho†	*p*
State audit	Sex			---	< 0.001^¶^
	Male	3.397 (0.977)	3.333 (1.333)		
	Female	3.164	3.000		
	Profession	(0.952)	(1.667)	---	< 0.001^§^
	Doctor	3.455 (1.045)	3.667 (1.667)		
	Dentist	3.406 (1.029)	3.667 (1.333)		
	Pharmacist	3.380 (1.008)	3.500 (1.333)		
	Nurse	2.991 (0.923)	3.000 (1.333)		
	Other health professional	3.214 (0.931)	3.333 (1.333)		
	Other profession	3.225 (0.864)	3.333 (1.333)		
	Units completed	---	---	0.076	(0.018) ^‡^
Knowledge	Sex			---	< 0.001^¶^
	Male	1.348 (0.581)	1.000 (0.667)		
	Female	1.648 (0.722)	1.333 (1.000)		
	Profession			---	< 0.001^§^
	Doctor	1.231 (0.441)	1.000 (0.333)		
	Dentist	1.188 (0.359)	1.000 (0.167)		
	Pharmacist	1.019 (0.077)	1.000 (0.000)		
	Nurse	1.524 (0.579)	1.333 (1.000)		
	Other health professional	1.700 (0.734)	1.667 (1.000)		
	Other profession	1.918 (0.831)	2.000 (0.667)		
	Units completed	---	---	−0.140	< 0.001
Drug quality	Age	---	---	−0.064	(0.046)
	Professional status			---	(0.039)^¶^
	Employed	2.693 (0.965)	2.667 (1.333)		
	Unemployed	2.986 (1.047)	3.167 (1.667)		
Drug substitution	Age	---	---	0.099	0.002
	Sex			---	(0.048)^¶^
	Male	3.872 (1.209)	4.000 (2.000)		
	Female	3.802 (1.076)	4.000 (2.000)		
	Marrital status			---	(0.048)^§^
	Single	3.685 (1.185)	4.000 (2.000)		
	Married	3.889 (1.096)	4.000 (2.000)		
	Divorced	4.012 (1.056)	4.500 (1.500)		
	Widowed	3.333 (1.155)	4.000 (2.000)		
	Profession			---	< 0.001^§^
	Doctor	4.343 (0.913)	5.000 (1.000)		
	Dentist	3.969 (1.177)	4.250 (1.500)		
	Pharmacist	2.139 (1.382)	1.500 (2.000)		
	Nurse	3.644 (1.101)	4.000 (1.500)		
	Other health professional	3.682 (1.117)	4.000 (1.500)		
	Other profession	3.782 (0.940)	4.000 (1.500)		
Fiscal impact	Profession			---	< 0.001^§^
	Doctor	2.622 (0.873)	2.667 (1.333)		
	Dentist	2.427 (0.963)	2.000 (1.000)		
	Pharmacist	2.611 (1.006)	2.667 (1.500)		
	Nurse	2.103 (0.798)	2.000 (1.000)		
	Other health professional	2.159 (0.812)	2.000 (1.000)		
	Other profession	2.151 (0.773)	2.000 (1.000)		

* for categorical variables, ** *IQR* Interquartile rage, †for ordinal or continuous variables, ¶ Wilcoxon rank-sum test, § Kruskal-Wallis rank test, ‡*p*-values in parentheses denote *p*-values< 0.05 who become statistically insignificant after application of the Bonferonni correction (corrected *p*-value = 0.007)

## Discussion

This cross-sectional study, carried out on students of the M.Sc. program in Health Management of the Hellenic Open University, aimed to assess their attitude, perception and knowledge regarding generic medications, using the ATTOGEN questionnaire. This is the first study to be conducted on a large number of students while using a validated questionnaire. The results indicate a mixed overall attitude of students towards generic medicines. Indeed, they did not feel quite trustful in generic medicines or in the abilities of Greek and EU authorities to address the increased pharmacovigilance mandates that generic substitution requires. Similarly, they were not convinced about the quality equivalence of generic and brand name medicines. On the contrary, they recognized the generic medicines’ positive fiscal impact to some extent. They also declared an increased level of knowledge and marked disagreement with drug substitution done by pharmacists. Other Greek studies involving physicians that were conducted before [25, 26] and after [27] the advent of the economic crisis confirmed these findings.

Further commenting on the results of this study, knowledge emerged as a powerful factor shaping the students’ attitude towards generics. Indeed, it seems rational to infer that high levels of information regarding generic medicines results in better knowledge about generics. This in turn leads students to increase their level of trust upon them and dissolve their skepticism, to recognize the quality equivalence of generics to branded products as well as their contribution to the reduction of healthcare costs and to the reallocation of resources and, therefore, to support drug substitution not only by doctors but also by pharmacists. Trust upon generics has been consistently reported as an important barrier to the increased use of generics [13]. Indeed, in this study, trust seemed to be highly correlated with all other ATTOGEN scales. In contrast, less informed students, students who were more skeptical towards generic medicines because of their lower quality or price felt that generic medicines were invented and promoted to resolve the financial crisis of social security institutions at the expense of citizens and did not acknowledge their positive fiscal impact to such an extent, compared to other students. The latter strong correlation has been confirmed by an older study on primary care patients [28]. Therefore, the myth that lower drug price equates to lower quality needs to be broken [29]. This study further suggests that students who believe in the state’s audit capabilities seem more reassured about the generic medicines’ quality, tend to feel less skeptical about them and to favor drug substitution not by only doctors but also by pharmacists.

Another important finding of this study is the distinct response pattern exhibited by pharmacists, compared to other professionals, which has never been studied to such an extent in the literature so far. In particular, pharmacists seemed to declare having a superior knowledge of generic medicines, to be more satisfied with the information they receive regarding this issue and to strongly believe in drug substitution, which is probably due to their superior knowledge in pharmacology. Strikingly, however, although this superiority would not justify any noticeable distinction between branded and generic medicines, comparatively to other professionals, pharmacists seemed to declare more often that they notice substantial differences between these two categories. Most importantly, despite their dual pharmaceutical and managerial academic background, they further argued more often than others that generics were invented and promoted in order to resolve the financial crisis of social security institutions at the expense of citizens. However, there seemed to be no statistically significant association between the “drug quality” or “trust” scale and profession, thus rejecting the existence of a true profession-dependent belief in inferiority of generics. This is consistent with a Czech study, where the vast majority of pharmacists stated that generic medicines are not inferior in terms of quality and side effects (83.9 and 88.8% respectively) [30]. In contrast, in another study evaluating New Zealand’s pharmacists’ view, knowledge and perceptions regarding generic medicines, Babar et al. conclude that, although pharmacists recognized the economic benefits of generic medicines in favor of the health system, concerns were raised regarding their quality, safety and effectiveness [31]. This is confirmed by the findings of an earlier Australian study on senior medical students and pharmacy pre-registrants [14].

Finally, the belief in generic medicines’ positive fiscal impact was not consistent in all respondents’ subgroups. Interestingly, doctors and pharmacists favor this opinion to a lesser extent, perhaps because of their knowledge that the price differences between originators and generics are still small in Greece [32] and that cases of high cost generic medicines exist [33].

This study bears certain limitations that should be addressed. Firstly, the study was conducted on students of the H.O.U. that may not be representative of the entire student population. On the other hand, these students belong to various age, economic and sociodemographic groups, because of the nature of the study environment (distance learning), which may somewhat increase the generalizability of the study’s results. Secondly, in this sample almost all students were employed, most of which in clinical practice as healthcare providers. This high employment rate is probably due to the distinct nature of the H.O.U. studies and perhaps, to the relatively high tuition fees for Greek standards. Although employment status was only associated with the “drug quality” scale, the magnitude of associations with other scales and their level of statistical significance may have been different among other student population samples. Finally, since the data collection form was filled online, we could not guarantee that some participants might have referred to any information sources to provide the correct answer, which could have underestimated the true lack of knowledge regarding generic medicines.

## Conclusions

This study demonstrated a mixed attitude of students regarding generic medicines. Knowledge emerged as an important factor shaping the students’ attitude towards generics. Among students, pharmacists exhibited a distinct response pattern. This study further stresses the need to address and correct health management students’ misconceptions and myths related to generics’ quality and utility. It is important that policymakers empower health professionals with detailed information about generic medicines, in order to ascertain the steady growth of their market position in the near future. Further studies are needed to confirm our findings and ascertain their generalizability to the entire Greek population.

## Additional file

Additional file 1: Dataset. (XLSX 304 kb)

## Abbreviations

ATTOGEN: ATtitude TOwards GENerics
HOU: Hellenic Open University
